# Altrenogest during early pregnancy modulates uterine glandular epithelium and endometrial growth factor expression at the time implantation in pigs

**DOI:** 10.1590/1984-3143-AR2020-0431

**Published:** 2021-05-28

**Authors:** Bruno Bracco Donatelli Muro, Diego Feitosa Leal, Rafaella Fernandes Carnevale, Mariana Andrade Torres, Maitê Vidal Mendonça, Denis Hideki Nakasone, Cristian Hernando Garcia Martinez, Gisele Mouro Ravagnani, Matheus Saliba Monteiro, André Pegoraro Poor, Simone Maria Massami Kitamura Martins, Priscila Viau, Cláudio Alvarenga de Oliveira, Raquel Vasconcelos Guimarães de Castro, Brendon Willian Bessi, Fabiana Fernandes Bressan, Lidia Hildebrand Pulz, Ricardo Francisco Strefezzi, Glen William Almond, André Furugen Cesar de Andrade

**Affiliations:** 1 Departamento de Reprodução Animal, Faculdade de Medicina Veterinária e Zootecnia, Universidade de São Paulo, Pirassununga, SP, Brasil; 2 Departamento de Zootecnia, Faculdade de Zootecnia e Engenharia de Alimentos, Universidade de São Paulo, Pirassununga, SP, Brasil; 3 Departamento de Medicina Veterinária, Faculdade de Zootecnia e Engenharia de Alimentos, Universidade de São Paulo, Pirassununga, SP, Brasil; 4 College of Veterinary Medicine, North Carolina State University, North Carolina, Raleigh, United States of America

**Keywords:** corpus luteum, endometrium, gilts, IGF-I, progesterone

## Abstract

This study evaluated the effects of supplying altrenogest from day 6-12 of pregnancy on the endometrial glandular epithelium, corpora lutea (CL) morphology, and endometrial and CL gene expression. A total of 12 crossbred females (Landrace × Large White) were used. The females were assigned to 4 treatments according to a random design with a 2 × 2 factorial arrangement, with two categories (sow or gilt) and two treatments (non-treated and treated with altrenogest). On day 6 of pregnancy, animals were allocated to one of the following groups: non-treated (NT, n = 6; 3 sows and 3 gilts), and (T, n = 6; 3 sows and 3 gilts) treated daily with 20 mg of altrenogest, from day 6-12 of pregnancy. All animals were euthanized on day 13 of pregnancy. All CLs were individually weighed, and their volume were determined. The endometrial glandular density (GD), mean glandular area (MGA), and vascular density (VD) were determined by histomorphometric and immunohistochemical analyses. Endometrium samples were collected and analyzed by qRT-PCR to evaluate the abundance of transcripts for VEGF and IGF-I. Females in the T group had higher MGA (*P* < 0.05) compared to the NT group. There was no effect of treatment on GD or VD for both experimental groups. Sows in the T group had augmented expression of IGF-I (*P* < 0.05). Progestagen had no detrimental effect on CL morphology. In conclusion, altrenogest improves the uterine environment during the peri-implantation period in pigs without compromising corpora lutea development.

## Introduction

In pigs, the peri-implantation period of accelerated trophoblastic elongation and attachment to the uterine surface relies on an intricate interplay between conceptuses (embryo/fetus and its associated membranes) and the uterine epithelium. During this phase, various inducible transcription factors are activated in response to hormone stimuli, resulting in the expression of a plethora of genes involved in conceptus development (Kridli et al., 2016). Progesterone (P4) plays a crucial role in pregnancy establishment, stimulating the secretion of molecules that modulate the uterine environment before implantation (Okrasa et al.*,* 2014). Among these, IGF-1 and VEGF, are highly expressed, exerting influence on conceptus growth and endometrial architecture (Waclawik et al., 2017). Circulating IGF-I concentration is positively related to fetal weight and is lower in intrauterine growth-restricted pig conceptuses (Wang et al., 2017). Uterine IGF-I increases trophectoderm mitotic rate (Lewis et al., 1992), being a limiting factor for the final size of the placental surface area (Geisert and Schmitt, 2002). Endometrial VEGF is a potent stimulator of angiogenesis and vascular remodeling during implantation and regulates vascular permeability (Welter et al., 2003). Of note, VEGF was found to be down-regulated in the placentae of intrauterine growth-restricted pigs (Chen et al., 2015).

These arguments sustain the notion that an adequate concentration of circulating P4 during the peri-implantation period would improve the uterine environment. Indeed, in bovines, supplemental P4 in early pregnancy affects endometrial gene expression (Forde et al., 2009), which may be the underlying cause of increased embryo development and higher pregnancy rate in cows (Pugliesi et al., 2016). Previously, Muro et al. (2020) demonstrated that sows supplemented with altrenogest from day 6-12 of pregnancy had heavier and larger embryos at 28 days of pregnancy. In pigs, there is limited information on the effects of progestagen provided during early pregnancy on the uterine environment.

Thus, this experiment tested the hypothesis that altrenogest provided from day 6-12 of pregnancy improves endometrial glandular development and increases endometrial gene expression of IGF-I and VEGF around the period of implantation in pigs.

## Methods

### Ethics committee approval

The experiment was performed according to the legal and ethical standards of the Ethics Committee for the Use of Animals of the School of Veterinary Medicine and Animal Science of the University of São Paulo (CEUA – 1168030817).

### Animals, housing and diet

The experiment was performed at the Swine Research Center of the School of Veterinary Medicine and Animal Science of the University of São Paulo, Brazil. A total of 12 crossbred females (Landrace x Large White), consisting of 6 sows (parity 1-3) and 6 gilts were used. Animals were checked for signs of estrus twice daily (08h00 and 16h00 hours) by fence-line contact with a mature boar and a back-pressure test. All animals were artificially inseminated with fresh diluted semen (3 × 10^9^ sperm cells; >80% motility) from the same boar. Gilts were inseminated at first signs of standing estrus and every 24 hours while still showing a standing estrus, totaling 2.3 inseminations. Sows (226.30 ± 3.33 kg BW, 14.25 ± 1.07 mm of backfat thickness and average weaning-to-estrus interval of 5 ± 1 day) were inseminated in the first estrus following a lactation period of 21 days at 12 hours after first signs of standing estrus and every 24 hours while ever the sows were in standing estrus, totaling 2.7 inseminations. Gilts were inseminated in the second observed standing estrus at approximately 240 days of age and 148.83 ± 1.86 kg BW. The second insemination was standardized as day 0 of pregnancy. All animals were allocated to individual pens and kept under similar nutritional and sanitary management. Females were fed 2.4 kg/d of a standard gestation diet (2930 kcal ME kg/d, 16% CP and 0.6% Lys) from day 0 to 13 of pregnancy.

### Experimental design

The females were assigned to 4 treatments according to a random design with a factorial arrangement of treatments (2 × 2), with two categories (sow or gilt) and two treatments (non-treated; NT, n = 6; 3 gilts and 3 sows, and treated with altrenogest; T, n = 6; 3 gilts and 3 sows). From day 6 to day 12 of pregnancy, the altrenogest was administered top-dressed individually with the morning feed, 20 mg of altrenogest/day (Regumate® - Merck Animal health, São Paulo, Brazil). Animals were euthanized on day 13 of pregnancy. The uterine horns were flushed with saline solution, and females were deemed pregnant when filamentous embryos were observed.

### Corpora lutea and progesterone measurements

The animals were euthanized at a local abattoir by stunning and exsanguination, and their genital organs were immediately collected. The ovaries were removed, and the number of CLs was counted on both ovaries. All CLs were individually dissected and weighed. The CL was considered as a sphere to determine its volume. The diameter from each CL was calculated by the average between width and length, measured with a digital caliper (Absolute Digimatic; Mitutoyo Sul Americana Ltda., Brazil).

Blood samples were collected by venipuncture on days 5, 6, 8, and 12 of pregnancy for serum P4 concentration measurements. The samples were centrifuged for 10 minutes at 1500 × g (centrifuge Excelsa II model 206; Fanem – São Paulo, Brazil) and stored in 2 mL microtubes at -20 °C for subsequent analysis.

The P4 concentration was obtained by the solid phase radioimmunoassay using a commercial kit (RIA PROGESTERONE – Beckman Coulter). The assays were performed according to the protocol provided by the manufacturer. A total of 48 samples were analyzed in one assay, and in duplicate. The sensitivity was 0.01 ng/mL, and the intraassay coefficient of variation (CV) ranged from 3.19% to 7.35%. The test used in this study to measure P4 does not detect altrenogest; therefore, the serum P4 detected in this test for altrenogest-treated females is mainly from CL. The P4 concentration by number of CL (P4/CL) in each treatment was considered as a variable in this study.

### Histology of uterine glandular epithelium

Three samples per animal of uterine tissue (approximately 4 cm^2^) were collected from different regions of the left uterine horn to assess the uterine glandular epithelium. The regions studied were: the utero-tubal junction (A1), the mid-portion (A2) and the utero-cervical junction (A3). After collection, all samples were fixed by immersion in 10% buffered formalin for 24 h and routinely processed for histology. Histological sections of 4 µm thickness were obtained and stained with hematoxylin and eosin, as previously described by Prophet et al. (1992). Three images per slide from each of the endometrial areas (A1, A2 and A3, total = 9 images/animal) were saved using the 4x objective (total area of each image = 7.45 mm^2^), were captured as “jpeg” files, using a microscope (Leica DM500) coupled with a high-definition camera (Leica ICCD50 HD). Glandular density (GD, glands/mm^2^) was determined by the number of glands divided by the area of the endometrium in the fields. Endometrial area was measured individually for each histological field, from the top of the luminal epithelium to the myometrium using the tool “freehand selections” of ImageJ® software. Mean glandular area (MGA, µm^2^), was evaluated as average size of each glandular structure. The average area of 50 glands per photomicrograph was measured using ImageJ® software. The same observer, in a blind evaluation, performed all the histological analyses.

### Endometrial vascularization

The immunohistochemistry technique was used to evaluate the vascular density of the endometrium. Histological sections adhered to silanized slides, dewaxed in xylene, rehydrated in graded alcohol followed by distilled water. Endogenous peroxidase was blocked for 30 minutes in 3% hydrogen peroxide solution. Antigen retrieval was performed with trypsin (Trypsin Enzymatic Antigen Retrieval Solution, ab970, Abcam), for 15 minutes, at room temperature. After incubation with 5% skimmed milk diluted in distilled water for 30 minutes to block non-specific protein binding, the slides were washed with Tris-buffered saline (TBS) with 1% Tween20 and incubated with a rabbit polyclonal primary antibody anti-Von Willebrand Factor antibody (Von-Willebrand Factor antibody – Abcam, Cod. Ab6994, 1:100), for 16 hours, in a humid chamber, at 4 °C. After washing with buffer, the samples were incubated with secondary antibody (Easylink ONE®, EasyPath, Brazil), following manufacturer instructions. For negative control, slides were incubated with rabbit IgG, at the same concentration, and processed simultaneously as those used for primary antibodies. Ten photomicrographs from the A2 endometrial area per animal were captured as “jpeg” files, using the 10x objective (total area for each image = 1.225 mm^2^). The average of ten photomicrographs was used to calculate the vascular density (VD, vessel/mm^2^of the endometrium), which was determined by the count of immunolabelled blood vessels divided by the area of endometrium in each image.

#### Quantitative Real-time PCR (qRT-PCR)

The qRT-PCR was performed according to Mesquita et al. (2014) and Machado et al. (2020) with minor adaptations, as described below. A sample of the endometrium with approximately 1 cm^2^ from the central region of the left uterine horn was collected for quantification of vascular endothelial growth factor (VEGF), the abundance of transcripts and their receptors I and II (VEGFR-I and VEGFR-II), and insulin-like growth factor (IGF-I) and its receptor (IGFR-I). Thus, 5 genes were evaluated in the endometrium. Additionally, the quantification of the LH receptor (LHR) transcripts in CL was performed. A sample for LHR gene quantification was composed of 4 fragments of approximately 1 cm from four CL per animal. The samples were stored in a freezer at -80 °C for further analysis. The forward (Fwd) and reverse (Rev) sequence of the primers were designed using the Primer Designing Tool (NCBI) using the porcine genome as reference or were retrieved from literature, and are showed in Table 1.

**Table 1:** Forward and Reverse primers of target genes used for real-time PCR gene quantification.

**Gene**	**5’- 3’ Forward**	**5’- 3’ Reverse**	**bp**	**Reference**
VEGF	TCACCAAGGCCAGCACATAG	GAGACGTCTGGTGCCCAAAA	163	XM_013977975.1
VEGFR-I	CACCCCGGAAATCTATCAGATC	GAGTACGTGAAGCCGCTGTTG	180	10.1016/j.mce.2008.04.020
VEGFR-II	GATGCTCGCCTCCCTTTGA	AGTTCCTTCTTTCAAGCGCCTACA	180	10.1016/j.mce.2008.04.020
IGF-I	CTCTCCTTCACCAGCTCTGC	GCCTCCTCAGATCACAGCTC	196	10.1016/j.mce.2014.01.023
IGFR	TCCTAGCACCTCCAAGCCTA	GTCTTCGGCCACCATACAGT	131	10.1016/j.mce.2014.01.023
LHR	GCCTCAGCCGACTATCACTC	AAGCATTTGCTGGTACGGTG	357	NM_214449.1
GAPDH-3	GTCGGTTGTGGATCTGACCT	ACCAGGAAATGAGCTTGACGA	221	NM_001206359.1

First, the mRNA was extracted with Trizol (#15596026, Thermo Fisher). Briefly, the Trizol was added to the samples, and after 5 minutes, chloroform was added for 3 minutes. The sample was centrifuged at 15000 × g for 15 minutes at 4 °C, and the translucid phase was separated into a new tube. An equal volume of isopropanol was added to the tube, and the sample was incubated at -80 °C for 2 hours. The sample was centrifuged, and 1 ml of 75% ethanol was added. After new centrifugation, the pellet was left to dry, and RNA quantification was performed using a NanoDrop™ 2000/2000 (Thermo Scientific™) spectrophotometer. The cDNA synthesis was performed using the High-capacity Reverse Transcription kit (Applied Biosystems, CA, USA), also according to the manufacturer’s instructions.

Relative quantification of the transcript levels was performed by using a 7500 Fast Real-time PCR System with PowerUP SYBR Green Master Mix (#A25777, Thermo Fisher). The reactions were performed at 95 °C for 15 minutes, and then 40 cycles at 95 °C for 15 seconds, 60 °C for 5 seconds and 72 °C for 2 minutes. A melting curve was performed for evaluation of specific amplification of the products, and all reactions were done in duplicate. The threshold cycle (Ct) values of the target genes were normalized by the mean of the housekeeping gene GAPDH. The fold changes were calculated using the 2^(-ΔCT)^ equation.

### Statistical analysis

In this experiment, completely randomized design with a factorial arrangement was used. The factor one was the category of females (gilt or sow) and factor two, the treatment used (treated with altrenogest and non-treated). Data were analyzed using the PROC GLM of SAS (v. 6.1; SAS Institute, Inc., Cary, NC). Each animal was considered one experimental unit. The LSD test was used to evaluate the treatment and category effect as well as their respective interactions. The data from progesterone concentration was analyzed by repeated measures. The data are presented as mean ± SEM, and differences were considered significant when P<0.05, and P<0.10 was considered a trend for the test of main effects.

## Results

### Corpora lutea and progesterone measurements

Females from T and NT had a similar CL number, with sows presenting more CL than gilts (Table 2). There was no effect (P > 0.05) of treatment or category for CL volume. The CL weight was not affected (P > 0.05) by treatment; however, there was an effect of the category (P < 0.05) for this variable, with gilts having heavier CL than sows (Table 2).

**Table 2:** Effects of altrenogest provided from day 6 to 12 of pregnancy on corpora lutea (CL) volume and weight on day 13 of pregnancy in sows and gilts1.

**Treatment**		**CL number**	**CL volume (mm^2^)**	**CL weight (mg)**
**Group**	**Category**			
Treated	Gilt	16.33 ± 1.76	457.07 ± 21.39	0.42 ± 0.02
Treated	Sow	29.00 ± 1.53	445.33 ± 11.94	0.33 ± 0.01
Non-Treated	Gilt	18.67 ± 1.86	448.85 ± 8.33	0.42 ± 0.01
Non-Treated	Sow	26.00 ± 5.03	459.23 ± 19.10	0.36 ± 0.01
Main effect				
Treated		22.67 ± 3.02	449.56 ± 10.81	0.36 ± 0.01
Non-Treated		22.33 ± 2.91	454.86 ± 11.57	0.38 ± 0.01
Gilt		17.50 ± 1.26^B^	452.68 ± 10.88	0.42 ± 0.01^A^
Sow		27.50 ± 2.45^A^	451.85 ± 10.96	0.34 ± 0.01^B^
P-value				
Group		0.912	0.668	0.336
Category		0.009	0.662	<0.001
Group x Category		0.389	0.812	0.151

^1^Data are presented as mean ± SEM. ^A,B^Means without a common superscript capital letter within column differ (P < 0.05).

**Figure 1:**
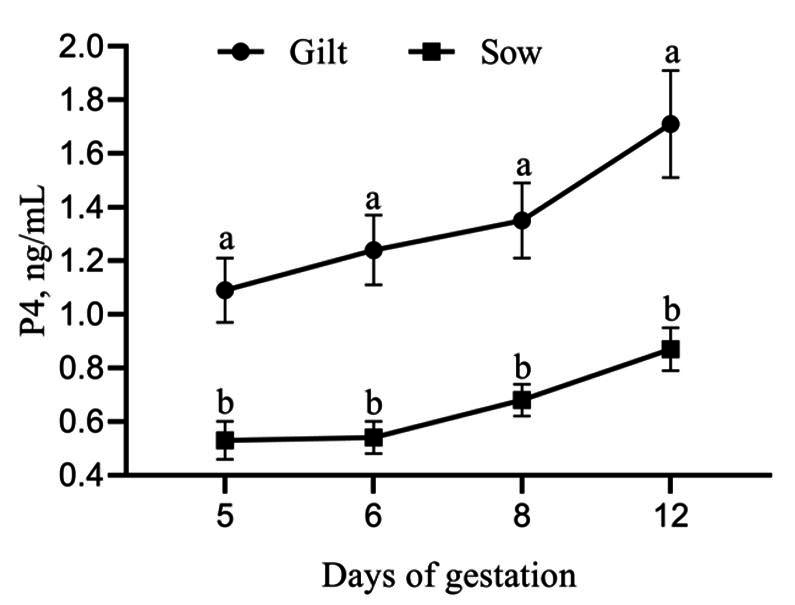
Progesterone concentration by CL number (P4/CL) in sows and gilts regardless of treatment. Different letters superscripts indicate significant differences (P < 0.05). Data are presented as mean ± SEM.

There was no effect (P > 0.05) of treatment for P4 concentration on day 5 of pregnancy and on the subsequent days of measurement (NT: 0.68 ± 0.10, 0.77 ± 0.17, 0.93 ± 0.19, 1.20 ± 0.21, on days 5, 6, 8 and 12, respectively; T: 0.80 ± 0.15, 0.84 ± 0.16, 0.94 ± 0.15, 1.19 ± 0.22, on days 5, 6, 8 and 12, respectively). However, the P4 concentration was influenced by category (P < 0.05), with gilts exhibiting higher P4 concentration concerning CL number (Figure 1).

### Endometrial glands and vascularization

**Figure 2:**
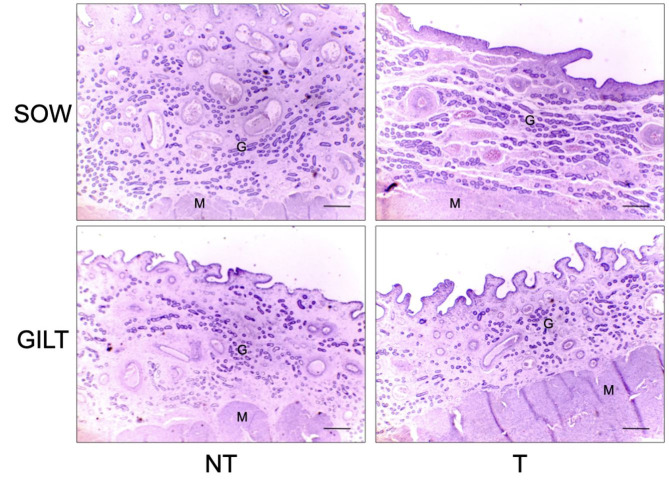
Histology of uterine glandular epithelium of swine female treated with altrenogest from day 6 to 12 of pregnancy. Representative photomicrographs of hematoxylin and eosin-stained uterine cross-sections of gilts and sows treated or non-treated (T and NT, respectively) on day 13 of pregnancy are shown. There was not a significant effect (P > 0.05) of treatment or category for glandular density (GD). Treated females had higher (P < 0.05) mean glandular area (MGA), irrespective of category. Sows also presented higher (P < 0.05) MGA compared to gilts, irrespective of treatment. M = myometrium; G = endometrial glands. Bars = 295µm.

No effect of treatment or category was observed for GD. The T group had higher MGA than the NT group (effect of treatment; P < 0.05) (Table 3; Figure 2). No effect of treatment (P > 0.05) was observed on endometrial VD in both categories. However, gilts presented higher VD than sows (effect of the category; P < 0.05).

**Table 3:** Effects of altrenogest provided from day 6 to 12 of pregnancy on endometrial glandular epithelium and vascular density on day 13 of pregnancy in sows and gilts.^1^

**Treatment**		**GD**	**MGA**	**VD**
**Group**	**Category**			
Treated	Gilt	120.60 ± 14.20	1072.05 ± 70.74	93.90 ± 17.48
Treated	Sow	102.63 ± 8.28	1916.06 ± 133.35	75.00 ± 10.72
Non-Treated	Gilt	116.30 ± 11.37	877.07 ± 63.47	109.17 ± 24.33
Non-Treated	Sow	112.18 ± 7.79	1431.09 ± 154.69	65.87 ± 4.53
Main Effect				
Treated		111.61 ± 8.27	1471.84 ± 122.18^a^	84.45 ± 10.09
Non-Treated		113.95 ± 6.44	1168.66 ± 106.76^b^	87.52 ± 14.70
Gilt		118.45 ± 8.84	979.69 ± 51.88^B^	101.53 ± 13.82
Sow		108.08 ± 5.65	1660.81 ± 115.32^A^	70.43 ± 5.59
P-value				
Group		0.740	0.045	0.861
Category		0.388	<0.001	0.091
Group x Category		0.481	0.744	0.465

GD = glandular density (glands/mm^2^); MGA = median glandular area (μm^2^/gland); VD = vascular density (vessels/mm^2^). ^1^Data are presented as mean ± SEM. ^a,b^Means without a common superscript lowercase letter within column differ (P < 0.05). ^A,B^Means without a common superscript capital letter within column differ (P < 0.05).

### Effects of progestogen on gene expression

**Figure 3:**
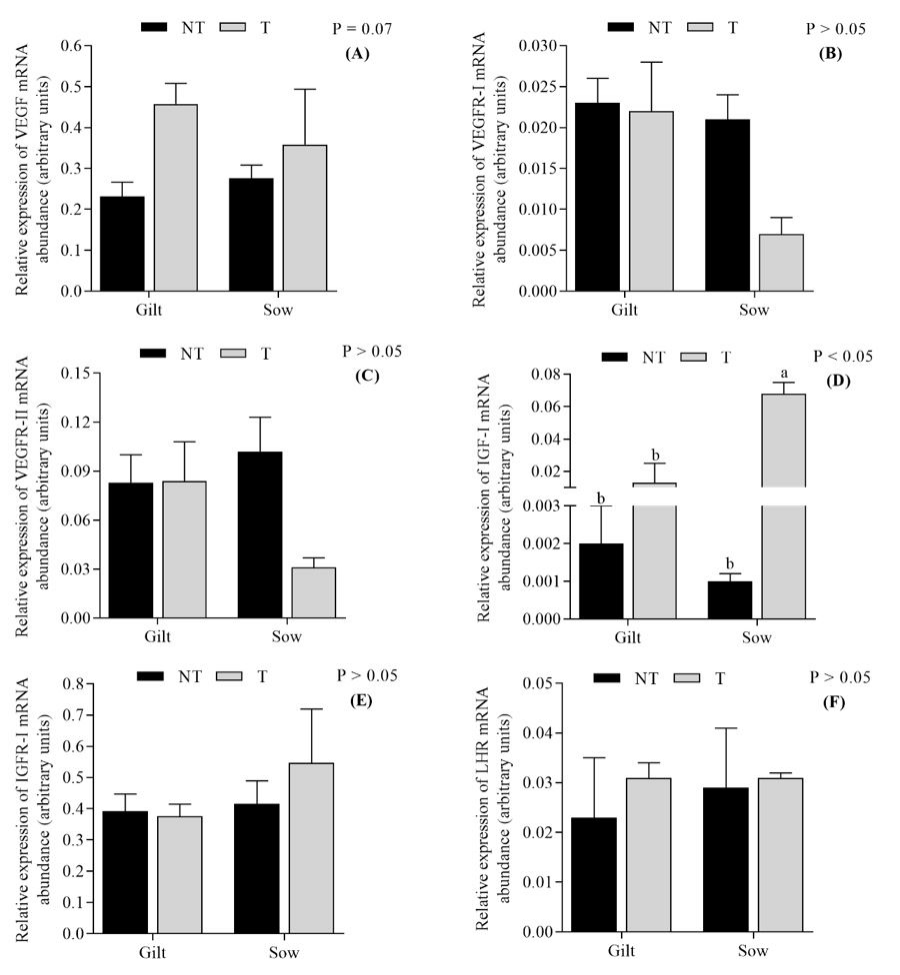
Relative expression of mRNA of VEGF (A), VEGF receptors I (B) and II (C), IGF-I (D), IGF-I receptor (E) on the endometrium and LH receptor (F) on corpora lutea in sows and gilts treated with altrenogest (T) from day 6 to 12 of pregnancy or non-treated (NT). Different letters superscripts indicate significant differences (P < 0.05).

The VEGF expression tended to be increased (P = 0.07) in the T group. There was no effect (P > 0.05) of the category for VEGF expression (Figure 3). No effect (P > 0.05) of treatment or category for VEGFR-I and VEGFR-II expression was observed. Sows in the T group presented augmented expression of IGF-I (P < 0.05) (Figure 3). For IGFR-I, neither treatment nor category affected (P > 0.05) its expression. There was no effect of treatment or category on the expression of LH receptor (LHR) in CLs.

## Discussion

The present study showed that sows treated with altrenogest had increased endometrial expression of IGF-I mRNA during early pregnancy, representing a 56-fold increase in its expression compared to non-treated sows.

There is mounting evidence to substantiate that IGF-I is highly relevant to peri-implantation events during early pregnancy in pigs (Jeong et al., 2014). Indeed, IGF-I protein in uterine flushings is highest on Day 12 of pregnancy, coinciding with the time of conceptus elongation (Geisert et al., 2001). Our results agree, in part, with Simmen et al. (1990), who found that gilts supplemented with P4 – through repeated intramuscular injections – presented increased uterine IGF-I gene expression. Albeit divergent between sows and gilts, our results demonstrated that altrenogest provided during early pregnancy could be used to increase endometrial secretion of IGF-I around implantation.

Another essential growth factor expressed at the time of implantation in response to P4 is VEGF. Endometrial VEGF plays a crucial role in angiogenesis, vascular remodeling, and permeability (Welter et al., 2003). Chen et al. (2015) showed that VEGF and its receptor VEGFR-1 are down-regulated in the placentae of intrauterine growth-restricted conceptuses. In the present study, altrenogest-treated females only tended to present increased endometrial VEGF expression, with no influence on endometrial angiogenesis. A possible explanation for our findings is that the short period of treatment was insufficient to affect endometrial vascular density. Besides, it is important to highlight that, possibly, the increase in the number of animals studied could lead to a more evident difference.

In our study, altrenogest treatment stimulated the hyperplasia of endometrial glands, regardless of category, probably affecting the microenvironment where first embryos develop. Bazer et al. (2012) reported that the glandular epithelium is responsible for synthesizing and secrete histotroph, critical to conceptus development. Likewise, Ravagnani et al. (2020) found increased uterine glandular area at 28 days of gestation in gilts with the previous estrus synchronized with 18 days of altrenogest treatment. Muro et al. (2020) also reported larger uterine glands at 28 days of pregnancy in gilts supplemented with altrenogest from day 6 to 12 of pregnancy. However, our results are not in agreement with Bailey et al. (2010), who did not observe hypertrophy nor hyperplasia in endometrial glands of ovariectomized gilts supplemented with long-acting progesterone. These conflicting results may be due to the stimulation of endometrial glandular epithelium by other ovarian and/or placental factors (Spencer et al., 1999; Young et al., 1990). Additionally, uterine glands of sows were more extensive in comparison with gilts, which may be explained by the fact that multiparous sows had already undergone glandular hyperplasia, most notably during the last trimester of previous pregnancies, making the uterine glandular epithelium primed for P4 (Sinowatz and Friess, 1983).

There is some evidence that porcine CL is not entirely “autonomous” until day 12 of the cycle, as previously believed (Ziecik et al., 2018). Inhibition of LH secretion with specific anti-LH antibodies (Szafranska and Ziecik, 1989) or gonadoliberin GnRH antagonist (Brüssow et al., 2001) during the early luteal phase of the estrus cycle reduced CL development and progesterone production. Therefore, considering the progestagen’s inhibitory effects on pituitary gonadotropins, studies intended to investigate the progestagen’s effects during early pregnancy should examine its effects on CL development.

The current study, sows and gilts treated with P4 from day 6-12 of pregnancy had no detrimental effects on luteal morphology. Additionally, the unaffected expression of LH receptors in the CL and progesterone levels in the serum corroborate with the morphological finds, thereby indicating that the altrenogest was not detrimental to CL function. The expression of progesterone receptors (PGR) in the uterine glandular epithelium until day 13 of gestation, together with the multitude of genes expressed in response to P4, endorse the unequivocal role of this hormone as the primary driver of early conceptus development in eutherian mammals (Bazer et al., 2012). Indeed, sows treated with progestogen from days 6-12 of pregnancy had larger and heavier embryos than non-treated sows without deleterious effects on embryo survival and pregnancy rate (Muro et al., 2020). Differently, Soede et al. (2012), in which gilts fed daily 20 mg of altrenogest from one to six days after the onset of estrus, for 18 days, had low embryo survival rate and pregnancy rate, possibly due to impaired luteal function.

Interestingly, in our study, gilts had heavier CL and higher concentrations of P4 to CL number. The physiological mechanism behind these results is yet to be determined. The highest concentration of P4 to CL number suggests that this finding might be associated with differences in ovulation rate (CL number) and/or differences in gilts and sows’ physiology instead of an effect caused by our treatment. Thus, further studies should be carried out to understand the mechanisms of altrenogest action that differ between sows and gilts.

In the present study and Muro et al. (2020) and Ravagnani et al. (2020), there are positive effects on the uterine endometrium by the administering altrenogest. Besides, with the present experiment results, it is possible to relate the impact on the more significant embryo growth found by Muro et al. (2020) to a higher expression of IGF-1 by the endometrium of the sows treated with altrenogest.

## Conclusion

In conclusion, the altrenogest provided from day 6 to 12 of pregnancy resulted in an increment of endometrial gene expression of IGF-I and uterine glandular hyperplasia without compromising CL development in sows.
